# Not only vaccine hesitancy, but also vaccination campaign hesitancy drives measles epidemics in conflict-torn eastern DR Congo

**DOI:** 10.1186/s13031-024-00569-6

**Published:** 2024-02-01

**Authors:** Daan Van Brusselen, Ali Heshima Dubois, Lucien Kandundao Bindu, Zakari Moluh, Yvonne Nzomukunda, Laurens Liesenborghs

**Affiliations:** 1 Médecins Sans Frontières (MSF), Masisi, Democratic Republic of the Congo; 2 Médecins Sans Frontières (MSF), Operational Center Brussels, Brussels, Belgium; 3 Department of Paediatric Infectiology, ZAS hospitals, Antwerp, Belgium; 4 Bureau Central de Zone (de Santé), Ministry of Health of North Kivu, Masisi, Democratic Republic of the Congo; 5 Department of Clinical Sciences, Institute of Tropical Medicine, Antwerp, Belgium; 6 Department of Microbiology, Immunology and Transplantation, KU Leuven, Leuven, Belgium; 7 University of Antwerp, Antwerp, Belgium

**Keywords:** Measles, Vaccination, Congo, Médecins Sans Frontières, Vaccine preventable diseases

## Abstract

The COVID-19 pandemic and vaccine hesitancy are not the only causes of the increase in measles cases in low- and middle-income countries. Measles epidemics, like the recent one in eastern DRC, are often quickly halted by mass vaccination in ‘easy to reach’ refugee camps. However, governmental and humanitarian actors fail to respond effectively in ‘hard-to-reach’ areas like Masisi, frequently limiting themselves to more accessible areas close to big cities.

## Article

Recently, several articles about measles outbreaks worldwide were published [1, 2]. The high measles case-fatality rate in young children in low- and middle-income countries (LMIC) and importance of timely vaccination were stressed once more [2]. We acknowledge the recent backslide in childhood immunization worldwide [3], and the resulting emergence of multiple measles outbreaks. However, we wish to remind the international medical community that the COVID-19 pandemic and vaccine hesitancy are not the only causes of the increase in measles cases in LMIC.

The international humanitarian organization Médecins Sans Frontières (MSF) has tackled measles outbreaks in the Democratic Republic of the Congo (DRC) for many years. Increasing tensions after the reappearance of several armed groups (especially M23), and the resulting large amount of internally displaced people (IDPs) (+-600.000) around Goma, have led to an upsurge in measles cases in the eastern part of the country this year.

In the first trimester of 2023 alone, the incidence of measles in the Masisi health zone (covering a population of 520,000, with an elevated rate of paediatric malnutrition) was 16 times higher than the yearly average (Table 1). However, like in many other regions in eastern DRC, reactive vaccination has only been implemented recently, because of security issues and poor accessibility of the area. In contrast, in a refugee camp in Kanyaruchinya (nearby the city of Goma, and easy to reach by health actors) a comparable increase in measles cases (Table 1) was effectively halted by massive vaccination in the beginning of the year (with almost no new cases since april).

**Table 1:** Measles cases, deaths and reactive vaccination in health structures supported by Médecins Sant Frontières Belgium in the province of North Kivu, Democratic Republic of the Congo

Masisi health zone (difficult to reach)						
Year / Period	2020	2021		2022		T1^*^ of 2023
Measles cases in MSF supported facilities	194	13		103		1684
Measles deaths in MSF supported facilities^**^	1	0		0		4
**Number of measles vaccines**	**0**	**0**		**0**		**0**
**Kanyaruchinya refugee camp (easy to reach)**						
**Trimester**	**T3** ^*^ **of 2022**		**T4** ^*^ **of 2022**		**T1** ^*^ **of 2023**	
Measles cases in MSF supported facilities	21		3		1411	
Measles deaths in MSF supported facilities^**^	0		0		6	
**Number of measles vaccines**	**13,232**		**0**		**108,949**	

^*^T1 = first trimester; T3 = third trimester; T4 = fourth trimester

^**^This constitutes only the number of measles deaths in MSF supported health structures; anecdotically we were informed of several more deaths in the community

Childhood vaccination remains one of the most cost-effective health interventions, avoiding > 40% of child mortality [4]. Unfortunately, in DRC, only 35% of children aged 12–23 months are fully vaccinated [5]. Because of the poor (access to) health care in the entire country, and even more so in the war-torn eastern DRC, there is no better way to prevent children from dying than by increasing vaccination coverage. However, governmental and humanitarian actors have failed to vaccinate ‘hard-to-reach’ areas like Masisi so far, often limiting themselves to more accessible areas close to big cities.

Armed conflict often disrupts societies and hampers access to health care [6]. Children who are affected by conflict suffer disproportionately from vaccine-preventable disease (VPD) outbreaks [7]. More than two-thirds of children who have not received their essential vaccines, live in countries that are either partially or entirely affected by conflict [6]. Even if health structures are not directly targeted in the Masisi region, the conflict increases food insecurity and forces people to flee into the bushes or gather in small towns where health infrastructure was already precarious before [7]. Crowding in a population with low vaccination coverage and a high malnutrition rate, inevitably leads to VPD outbreaks (like measles) [6, 7].

Most current vaccination strategies focus on refugee camps, often failing to reach IDPs in areas that are more difficult to reach. There is, therefore, an urgent need to include more remote populations outside of refugee camps in reactive vaccination strategies [6]. However, such efforts are demanding and less visible to the outside world than large-scale vaccination campaigns in refugee camps near accessible cities. Such campaigns in remote areas are, therefore, seldom a priority for national and international organizations, but remain crucial in preventing VPD outbreaks.

It is of utmost importance to uphold and strengthen international treaties that protect essential medical services (like vaccination) during conflict, also in remote areas, not just in theory but in practice [6]. In North-Kivu, we believe that it is crucial to negotiate a humanitarian corridor with the belligerent parties to allow catch-up vaccination and health promotion [7]. The use of new technologies could facilitate such an effort [6]: one could for example map populations with satellites or explore the possibility of drones to deliver vaccines when roads are less accessible.

Given the exceptional transmissibility of measles, and the vulnerability of malnourished children, a tailored vaccination approach in eastern DRC, targeting the most vulnerable (also in remote areas) in case of an outbreak, is absolutely needed. Security risks need to be tackled (e.g. by a humanitarian corridor, negotiated with the different belligerent parties), and logistical challenges have to be overcome (e.g. through the use of modern technology) by stakeholders like MoH, the UN and iNGOs.

## Data Availability

No datasets were generated or analysed during the current study.

## Abbreviations

LMIC: low- and middle-income countries
DRC: Democratic Republic of the Congo
IDPs: internally displaced people
MSF: Médecins Sans Frontières
VPD: vaccine preventable diseases
MoH: Ministry of Health
iNGO: international Non-Governmental Organization
UN: United Nations
